# The concept of informal care: ambiguities and controversies on its scientific and political uses

**DOI:** 10.3389/fsoc.2023.1195790

**Published:** 2023-07-18

**Authors:** Sofia Alexandra Cruz, José Soeiro, Sara Canha, Valentina Perrotta

**Affiliations:** ^1^Faculty of Economics, University of Porto, Porto, Portugal; ^2^Faculty of Arts, Institute of Sociology, University of Porto, Porto, Portugal; ^3^Centre for Research in Anthropology (CRIA), University Institute of Lisbon (ISCTE), Lisbon, Portugal; ^4^Faculty of Social Sciences, University of Republic of Uruguay, Montevideo, Uruguay

**Keywords:** informal care, unpaid care, scientific uses, political uses, public policies, gender inequality

## Abstract

Starting from an analysis of the scientific and political uses of the concept of informal care, this paper raises questions and launches the debate on the causes and effects of its uses. Recognizing the diversity and the contradictions found across the use of the term, it explains how its predominant use in Europe can be problematic. First, although it is widely recognized that care is provided primarily by women, this gender dimension is not emphasized in a concept that obscures the sexual division. Second, it does not render explicit that informal care is work, despite being unpaid. Third, the allusion to informality is likely to generate confusion with informal employment of care workers. Finally, studies often focus exclusively on care provided by family members, without distinguishing the spaces in which the work takes place and the social relationships it involves, namely the family or community. In Europe, where documents from (non)governmental organizations focus mainly on long-term care related to demographic aging, it is the care crisis of formal care provision systems, faced with financial fragility, reduction in funds and insufficient supply to meet the demand, that brings informal care to the political and scientific agendas. This paper argues that it is necessary to define conceptual boundaries that allow international studies on the dimension and value of this care work to be compared. It also advocates the importance of making visible that this is work, unpaid and female-dominated, since this view supports action guidelines more focused on social transformation and empowerment.

## Introduction

The scientific literature addressing care and the role of informal care has gained prominence in social sciences, particularly in analyses of long-term care systems and the relationships between formal and informal care (Greve, 2017; Verbakel, 2018; Barczyk and Kredler, 2019; Ariaans et al., 2021; Da Roit, 2021; Albuquerque, 2022; Tinios et al., 2022), care and welfare regimes and policies (Anttonen and Sipilä, 1996; Bettio and Plantenga, 2004, 2008; Ferrera, 2012; Torres et al., 2012; Albertini, 2014; Frericks et al., 2014; Batthyány, 2020), as well as the economics of care (Zelizer, 2011), the role of care in social reproduction (Bhattacharya, 2017) and the ethics of care (Tronto, 2013).

The current context of demographic, social and labor transformations has given care greater public visibility and put pressure on the reorganization of public care policies, which can be seen in the growing production of European, Latin American and Caribbean international reports and recommendations on long-term care (IACO, 2018; Spasova et al., 2018; EC, 2021a; Rocard et al., 2021; UN Women and ECLAC, 2021; ECLAC, 2022; PE, 2022) and, in particular, on informal Care (Zigante, 2018; EC, 2021b; Rocard and Llena-Nozal, 2022). On the other hand, changes in gender relations have given visibility to the naturalization and accountability of women as careers. These phenomena, together with the shortage of accessible and good quality public care services, have raised the debate on the sustainability of social protection systems, constituting what has been called the care crisis (Tronto, 2005; Orozco, 2006; Ezquerra, 2012; Fraser, 2017; Batthyány and Sol Sánchez, 2020; Dowling, 2022).

The study of the specific role of informal care has benefited from approaches that seek to locate it in a care diamond (Razavi, 2007), consisting of the State, the market, the community and the family. The social relations of informal care have also been viewed from an angle of the circuits of care (Guimarães, 2020), particularly in two of them: care as obligation, within the framework of family responsibility, and care as help, based on group or community reciprocity (Guimarães, 2020; Guimarães and Vieira, 2020). The reference to informal care refers to the provision of care to people in situation of dependency by family members, friends and neighbors (Lage, 2005; EC, 2021b). A comprehensive concept of care and provision of care (Laugier and Paperman, 2009; Molinier, 2013) makes it possible to capture the heterogeneities that differentiate classes and social groups in this provision (Kergoat, 2016) and to identify distinct modalities of social organization of care according to national contexts (Hirata, 2021; Aranco et al., 2022). This asymmetrical combination in each national reality of the different vertices of the care diamond coexists with common modalities of devaluation of informal care.

This article aims to critically analyse the use of the concept of informal care in academic and non-academic vocabulary. Recognizing the diversity and even the contradictions present in its uses, we argue that this concept is problematic in four dimensions: it makes the gender cut and sexual division invisible; it does not render explicit that we are dealing with work, regardless of its unpaid nature; the reference to informality may lead to confusion with the informal employment of care professionals; it does not distinguish the spaces in which this work takes place and the social relationships involved, namely the family or community.

## Informal care: scientific and political uses

The concept of care is multidimensional and constructed from distinct disciplinary, legislative and political frameworks, which potentiates considerable ambiguity and controversy (Glenn, 2016; Durán, 2018; ILO, 2018; Borgeaud-Garciandía and Guimarães, 2020; Fraser, 2020; Hirata, 2021; Tur-Sinai et al., 2022). Care work encompasses direct, personal and relational care activities, as well as indirect care activities (ILO, 2018). The former refers namely to support activities provided to people in situation of dependency, while the latter encompasses organization, preparation, tidying and cleaning tasks. The provision of this care can be paid or unpaid, have a short, medium or long-term duration, and take place within the family, within non-market relations of mutual help among friends or community members, and in professional relationships within the labor market (EC, 2021a; Jegermalm and Torgé, 2021; Rocard and Llena-Nozal, 2022).

This ILO (2018) understanding of the broad sense of care, although advocated by other organizations (Eurofound, 2020; EC, 2021a), is not always shared between researchers and organizations particularly with regard to informal care (Rutherford and Bu, 2018). According to IACO^*^ (2018), informal care is defined as unpaid care provided by family members, neighbors, friends or significant others who play the role of career to support someone with declining physical capacity, debilitating cognitive condition or life-limiting chronic illness. According to this perspective, care is defined from a relational dimension of support due to illness, disability and impairment, which implies a responsibility toward someone's life and wellbeing, and does not explicitly include non-relational tasks, namely domestic tasks. The diversity of conceptualizations about informal care is reflected in the guidelines adopted by different countries (Batthyány et al., 2017; Hirata, 2020; Araújo and Soeiro, 2021; Redondo and Benencia, 2021; Failache Mirza et al., 2022).

Since most informal care is performed by family members, mainly by women, the concept is often limited to particular types of relationships, namely family and household relations. Most of the literature focuses on family relationships, the studies on marital and filial relationships in the provision of care being dominant (Wagner and Brandt, 2018; Uccheddu et al., 2019; Bertogg and Strauss, 2020; Heger and Korfhage, 2020; Mazzotta et al., 2020). There are few studies that seek to examine other spheres of relationships in the provision of care (Larkin et al., 2019) and care within the community, which contributes to the dominant understanding of informal care as belonging to the universe of the family.

Most literature on care regimes and reproductive work understands what has been called informal care as unpaid work historically made invisible, being part of a widespread problem of devaluation of care work and gender inequality. By contrast, the literature on long-term care (LTC) systems in a context of demographic aging places the emphasis not on the recognition of informal care as unpaid work, but on the recognition of its social value and its importance for the sustainability of care systems (EC, 2021a; Tinios et al., 2022). Studies in the areas of health and social policies and aging analyse different aspects associated with informal care—the impacts on health (Verbakel, 2018; Zigante, 2018; Skinner and Sogstad, 2022), the role of gender (Uccheddu et al., 2019; Bertogg and Strauss, 2020; Skinner and Sogstad, 2022), the socio-economic inequalities (Bertogg and Strauss, 2020; Verbakel et al., 2022), the variation in the educational levels of informal careers (Lera et al., 2020; Rodrigues and Ilinca, 2022; Rostgaard et al., 2022).

## Informal care and inequalities: labor market, families and state

In addition to population aging, the rising retirement age (Starr and Szebehely, 2017; Mazzotta et al., 2020; Lam et al., 2022) has also caused pressure on informal caregivers. Most working-age informal careers combine care with paid work, but the employment rate declines with the intensity of care provided (EC, 2021a). Several recent studies have addressed the challenges and impact of care responsibilities on employment (Burch et al., 2019; Moussa, 2019; Clancy et al., 2020; Spann et al., 2020; Charmes, 2022; Lam et al., 2022). The impact of different labor market relationships on care is less studied (Mazzotta et al., 2020; Koreshi and Alpass, 2022). Different studies address the role of gender in the type of care provided and the consequent impact in jobs and earned income (Heger and Korfhage, 2020; Hsu et al., 2020; Peña-Longobardo et al., 2021; Hanson, 2022). The growth in the number of studies on employment and informal care shows a greater concern to support informal caregivers and help them stay in employment, and less concern to reduce their burden through redistributing care. Although the reduction and redistribution of care are key aspects in the European and international recommendations (ILO, 2018; Women, 2018; European Parliament, 2022), the vast majority of studies referred to focus on the impacts and promotion of informal care as co-producers of wellbeing in care systems and adopt a gender-blind approach. With an overriding understanding of informal careers as “genderless” family members, gender inequality is frequently invisibilised. Even though the impact of policies on gender relations is present in some studies (Szebehely and Meagher, 2018; Eggers et al., 2020; Hansen and Dahl, 2021), other types of inequalities, particularly socio-economic and ethno-racial, are little addressed (EC, 2021b).

Informal careers do not always perceive themselves as such, often because they consider the assistance provided as something that is expected within their social and family context (Rocard and Llena-Nozal, 2022). In this sense, it is important to recognize that informal careers are not a homogenous group as they have different burdens and responsibilities (Jegermalm and Torgé, 2021). Despite being a fundamental pillar of LTC in most countries, with consequences and costs for both careers and care recipients, as well as for the care sector in general (Ribeiro et al., 2021; Tur-Sinai et al., 2022; Charalambous, 2023), informal care is often overlooked, which makes the exercise of quantifying it difficult (Rutherford and Bu, 2018; Cès et al., 2019; Tur-Sinai et al., 2020). Several studies have endeavored to assess the economic value of these tasks provided as part of family ties, often unrecognized although they have been increasing (Hoefman et al., 2018; Oliva-Moreno et al., 2019; EC, 2021a; Ekman et al., 2021; White et al., 2021). Male and female time-use surveys (Rutherford and Bu, 2018; Folbre, 2021; Ribeiro et al., 2021) rightly highlight the need to consolidate definitions and classifications in order to build more coordinated and comparable approaches. Thus, the exercise of quantifying the economic value of this type of care does not appear to be a simple task, clearly due to the different methodological approaches used (Hanly and Sheerin, 2017; Durán, 2018; White et al., 2021; Perista and Perista, 2022).

The COVID-19 pandemic has expanded the attention and the number of studies on pandemic impacts on LTC systems (Rocard and Llena-Nozal, 2022) and on informal care (Dugarova, 2020; UN Women, 2020a,b; Eurocareers/IRCCS-INRCA, 2021; Lorenz-Dant and Comas-Herrera, 2021) has increased, mainly nationwide studies (Chan et al., 2020; Moré Corral, 2020; Phillips et al., 2020; Cohen et al., 2021; Rodrigues et al., 2021; Madia et al., 2023). They highlight the insufficiency of measures to support informal careers (Lorenz-Dant and Comas-Herrera, 2021) and the lack of data that would allow better knowledge of the phenomenon (Rocard and Llena-Nozal, 2022). Its impacts on gender inequality have received attention, including the increased workload of care for women, and there is a growing emphasis on the need to value care work (Bahn et al., 2020; Power, 2020; Seck et al., 2021; Camilletti and Nesbitt-Ahmed, 2022) and to address the intersection of gender, race and class inequalities (Lokot and Amiya, 2020; Osorio-Parraguez et al., 2022). A clear distinction in the approach to care according to the type of concept used is expressed in this literature. The concepts *informal care/family care* are used in the literature that underlines the social value of care and the need to support these careers as co-producers in the care system. The concept *unpaid care* predominates in the literature that emphasizes the inequalities in the provision of care and that explicitly recognizes the value of care as work. That is the predominant approach in Latin America, in which research has sought to identify the different unpaid activities as components that contribute to social wellbeing in the same way as paid work. In this process of recognition and visibility, care work has begun to gain prominence among other types of unpaid work (Aguirre et al., 2014; Batthyány, 2020). It should be noted that the main distinction made in feminist academic studies that address care provision has been whether it is paid or unpaid. They adopt a political stance of transformation, based on denouncing the impact on women's rights of taking on the majority of unpaid care work, particularly those with lower socio-economic status (Batthyány et al., 2017).

## Informal and formal care: a network of interactions

The analysis of the role of informal care has been made in the light of different care and welfare regimes, permeable to movements of defamiliarization and re-familiarization (Le Bihan et al., 2019; Da Roit, 2021; Cheneau and Fargeon, 2022). When these regimes are compared, it is possible to distinguish various models of interaction between informal care, with a family and community nature, and formal care, in the form of market services or public provision.

Comparative studies have highlighted national differences in the institutional variations of the welfare mix, the prevalence of health or social care and their articulation, the role of the family and community, and the uneven development of social responses for unpaid careers (Emilsson, 2009; Brimblecombe et al., 2018; EC, 2021a; Hirata, 2021). Some studies suggest that the incidence and intensity of informal care is negatively correlated with formal care provision (Barczyk and Kredler, 2019; EC, 2021a; Hollingsworth et al., 2022). Others observe complementarity regarding formal home care and informal care (Rapp et al., 2022; Tinios et al., 2022). Verbakel (2018) notes that the generosity of formal care varies the intensity of care, but that informal care is always present. Courbage et al. (2020) draw attention to the fact that the effect of public benefits on informal care depends on the typology of public coverage for LTC. In response to authors who considered policies that support extrafamily care as defamiliarising and policies (or lack thereof) that promote informal care provision by relatives as familiarizing (Leitner, 2003, 2014; Saraceno, 2010, 2016), different authors have argued that these represent two different types of policies that can vary relatively autonomously from each other, that we can expect different combinations of both types of policies, and that they will have varying effects on gender equality (Eggers et al., 2020) and on socio-economic inequalities (Verbakel et al., 2022). The disparate findings on complementarity or substitution between informal care and formal care of different types can be justified by the use of different variables of analysis (Verbakel, 2018; Barczyk and Kredler, 2019), and cultural factors may also contribute to explaining cross-national differences in people's care behavior (Spann et al., 2020; Tinios et al., 2022).

International comparison of data on the number of informal careers of people with LTC needs does not allow for unambiguous information. This difficulty becomes explicit when comparing, for example, the results of the European Health Interview Survey, the European Quality of Life Survey and the Study on Health and Aging in Europe, which show different results for the same countries (Tur-Sinai et al., 2022). In addition to the difficulty in measuring the real universe of informal careers, there is the ambiguous place they occupy in public policy: they are sometimes perceived as a resource of care policies, sometimes as co-producers of care, and sometimes as co-beneficiaries of care policies (Cheneau and Fargeon, 2022).

The role of informal careers for LTC policies has been internationally highlighted (Fiest et al., 2018; UNECE, 2019). In the European framework, reference to care first appeared in the context of health and childhood and, in recent decades, long-term care and the promotion of gender equality (di Torella and Masselot, 2020). In 2022, a Resolution for a common European action on care was adopted (European Parliament, 2022) in which, among other actions, the European Commission is urged to present a status and support for informal careers, and Member States to consider formalizing informal care. The Latin American and Caribbean region is discussing the implementation of national care systems as a result of the positioning promoted by the feminist movement together with the academia and the various commitments made by States in the regional gender agenda derived from the Regional Conference on Women of the Economic Commission for Latin America and the Caribbean (CEPAL, 2021, 2022).

## Public policies for informal careers: framings and limitations

The justification for the public policy measures on informal careers has been based on the need to mitigate the negative consequences of informal care, namely those related to the risk of poverty and physical and mental health deterioration (Bom et al., 2019; Kim, 2020; EC, 2021a; European Parliament, 2022) as well as those that have to do with employment, absenteeism, abandonment and absence from the labor market, difficult re-entering into that market, the need to promote a balance between care and work that results in extending careers (Bauer and Sousa-Poza, 2015; OECD, 2019; EC, 2021a; Grünwald et al., 2021). For example, the European Directive on work-life balance for parents and careers (Directive (EU), 2019), states that each worker should have the right to careers' leave of five working days per year, should be able to adapt their working schedules to their needs and preferences, and request flexible working arrangements, to remain in the work force. The importance of the category of informal careers in public policies is also the result of social mobilization of this group (Poch, 2017; Soeiro and Araújo, 2020).

Among the main policies on informal careers the following were identified: their legal recognition within the framework of care systems; direct cash benefits to careers or indirect care allowances, through the recipients of care and tax benefits; paid and unpaid leave and flexitime measures for workers-careers; psychological support and support groups; training and capacity building; assistive technologies; services provided to the people cared for as forms of support and redistribution of the work of informal careers, particularly home support and carer respite by sending the person cared for to long-term care facilities, under the health systems, social support structures or local authorities (Brimblecombe et al., 2018; Spann et al., 2020; Da Roit, 2021; EC, 2021a; IACO, 2021; Koreshi and Alpass, 2022). Policy measures differ according to national reality, namely regarding: the scope of the concept of informal carer, who may only be someone with family ties (Spain, Portugal, Denmark) or include other ties (Australia, France, Germany, UK, Finland); the duration of unpaid leave; the existence of paid leave, its duration and scope (only family members or not); the type of support equipment available. There has been important debate on referral criteria and how selective is the access (namely through determining the dependency of the person cared for or the socio-economic status of careers and dependent people); the integration between systems and the coherence of care policies; and the impact of the economic and pandemic crises on LTC (Da Roit, 2021; EC, 2021a; IACO, 2021; Cheneau and Fargeon, 2022).

Based on the literature on informal care, we identified three critical aspects in the uses of the concept in public policies. First, the porousness and blurring of the boundaries of the concept allows for very different operational definitions, which makes it difficult to compare international studies on the dimension and value of this type of work. Second, the choice of the term “informal care,” rather than “unpaid care work” mainly done by women, may render unequal care regimes natural and provide a basis for action orientations focused on individual empowerment, self-care and training, rather than on social transformation. Third, there is a contradiction between the apparently broader description of informal care and its operationalization for the purpose of access to policies supporting careers. These are often based on a narrow concept of informal care that only recognizes family care. In addition to disregarding community care, they may contribute to concealing the dynamic that reinforces (re)familiarization currently present in the social organization of care.

As we have seen, the concept of informal care is scientifically unstable and its use in public policies is delicate, particularly because it does not allow us to distinguish what is intended to be transformed as regards its informality. If we recognize the existence of this unpaid care and work to build its capacity and to make the conditions of informal careers legal, but do it without considering its remuneration or the disproportionate distribution of this work by gender, a reproductive effect on inequalities is to be expected. On the other hand, aspects such as the level of wages or the working conditions are also frequently missing when formal care, that is care provided by professionals, is referred. Concepts should make it possible to describe and understand the social phenomena, as well as to contribute to their transformation. The formal/informal binomial, as a useful category when thinking about care and classifying it, tends to obscure central dimensions of the phenomenon, rather than elucidate them. In this paper, we intend to contribute to the questioning and critical overcoming of this binomial by the research and intervention agenda in this area.

## Author contributions

All authors listed have made a substantial, direct, and intellectual contribution to the work and approved it for publication.
